# GLP-1 Receptor Agonists, Fertility Restoration, and Reproductive Safety in Women of Reproductive Age: A Narrative Review

**DOI:** 10.3390/jcm15093204

**Published:** 2026-04-22

**Authors:** Malak Moones Abedi, Mohamedanas Mohamedfaruk Patni, Arshiya Nasreen Bint Shajahan, Rajani Dube, Liyan Khadeeja, Ibrahim Alabid, Ahmad Kharoufeh, Subhranshu Sekhar Kar, Biji Thomas George, Shadha Nasser Bahutair, Thilakavathy Pandurangan

**Affiliations:** 1 RAK College of Medical Sciences, Ras Al Khaimah Medical and Health Sciences University, Ras Al Khaimah P.O. Box 11172, United Arab Emirates; malak.abedi0320@gmail.com (M.M.A.); arshiya.21901039@rakmhsu.ac.ae (A.N.B.S.); liyan.21901020@rakmhsu.ac.ae (L.K.); ibrahim.21901097@rakmhsu.ac.ae (I.A.); ahmad.21901103@rakmhsu.ac.ae (A.K.); 2 Department of Community Medicine, RAK College of Medical Sciences, Ras Al Khaimah Medical and Health Sciences University, Ras Al Khaimah P.O. Box 11172, United Arab Emirates; 3 Department of Obstetrics and Gynecology, RAK College of Medical Sciences, Ras Al Khaimah Medical and Health Sciences University, Ras Al Khaimah P.O. Box 11172, United Arab Emirates; shadha@rakmhsu.ac.ae; 4 Department of Pediatrics and Neonatology, RAK College of Medical Sciences, Ras Al Khaimah Medical and Health Sciences University, Ras Al Khaimah P.O. Box 11172, United Arab Emirates; subhranshu.kar@rakmhsu.ac.ae; 5 Department of General Surgery, RAK College of Medical Sciences, Ras Al Khaimah Medical and Health Sciences University, Ras Al Khaimah P.O. Box 11172, United Arab Emirates; biji@rakmhsu.ac.ae; 6 Department of Microbiology, RAK College of Medical Sciences, Ras Al Khaimah Medical and Health Sciences University, Ras Al Khaimah P.O. Box 11172, United Arab Emirates; thilakavathy@rakmhsu.ac.ae

**Keywords:** GLP-1 receptor agonist, semaglutide, liraglutide, pregnancy, fertility, preconception counseling

## Abstract

**Background/Objectives:** Glucagon-like peptide-1 receptor agonists (GLP-1RAs) are increasingly used for the management of obesity and type 2 diabetes, particularly among women of reproductive age. Emerging evidence suggests potential effects on ovulation, fertility, and pregnancy outcomes. This narrative review aims to synthesize current evidence on the reproductive safety of GLP-1RAs, with a focus on their implications for conception, unintended pregnancy, and maternal–fetal outcomes. **Methods:** A narrative literature review was conducted using PubMed and relevant bibliographic sources to identify studies published between 2020 and 2025. The search included clinical trials, observational studies, registry data, case reports, and selected preclinical evidence. Studies addressing reproductive outcomes, including ovulation, fertility, pregnancy exposure, and fetal safety, were included. Evidence was synthesized descriptively in accordance with recommended approaches for narrative reviews. **Results:** Available evidence indicates that GLP-1RAs may improve ovulatory function and menstrual regularity, particularly in women with obesity or polycystic ovary syndrome, potentially increasing the likelihood of conception. However, human data on pregnancy exposure remain limited. While current evidence does not consistently demonstrate a strong teratogenic signal, findings are based on small samples and heterogeneous study designs. Concerns persist regarding unintended pregnancies due to improved fertility and the absence of robust safety data during early gestation. **Conclusions:** GLP-1RAs present a complex clinical scenario in women of reproductive age, with potential benefits for metabolic and reproductive health but uncertain safety during pregnancy. Clinicians should exercise caution, provide appropriate contraceptive counseling, and carefully weigh the risks and benefits when prescribing these agents. Further large-scale, prospective studies are needed to clarify reproductive safety and inform evidence-based clinical guidelines.

## 1. Introduction

The global burden of obesity and type 2 diabetes mellitus has increased substantially over recent decades, affecting a growing proportion of women of reproductive age [1,2,3]. In parallel, conditions such as polycystic ovary syndrome (PCOS), which are closely linked to metabolic dysfunction, remain highly prevalent and represent a leading cause of anovulation and subfertility [4,5,6]. These intersecting metabolic and reproductive health challenges have important implications for both individual patient outcomes and broader public health, particularly in settings where delayed childbearing and lifestyle-related risk factors are increasingly common [2,4,7].

Glucagon-like peptide-1 receptor agonists (GLP-1RAs) have emerged as an important therapeutic option for the management of obesity and type 2 diabetes, with well-established effects on weight reduction, glycemic control, and cardiometabolic risk factors [8,9,10]. Beyond their metabolic benefits, there is growing interest in their potential influence on reproductive physiology [11,12]. Improvements in insulin resistance, weight, and hormonal balance may contribute to the restoration of ovulatory function and menstrual regularity, particularly among women with PCOS [5,11,12,13]. While these effects may represent a therapeutic advantage, they also introduce clinically relevant considerations regarding fertility, including the possibility of increased rates of spontaneous conception [11,14].

At the same time, the use of GLP-1RAs in women who may conceive raises important safety concerns [14,15,16]. Evidence regarding their effects during early pregnancy remains limited, and current recommendations generally advise caution or discontinuation prior to conception [15,16,17]. The potential for unintended pregnancy due to improved fertility, combined with insufficient human data on fetal safety, creates a complex clinical scenario that requires careful risk–benefit assessment [14,16,18]. This is particularly relevant in routine clinical practice, where these agents are increasingly prescribed for weight management in younger populations [8,9,14].

Despite the expanding use of GLP-1RAs, the available literature on their reproductive effects remains fragmented, with findings derived from clinical trials, observational studies, case reports, and preclinical research [11,14,18]. Existing evidence varies in scope and methodological rigor, and there remains a lack of consolidated synthesis focused specifically on reproductive-aged populations and clinically meaningful outcomes [14,18].

Accordingly, the objective of this narrative review is to synthesize current evidence on the reproductive implications of GLP-1 receptor agonists, including their effects on ovulation and fertility, the risk of unintended pregnancy, and available data on pregnancy exposure and maternal–fetal outcomes. By integrating findings across different study designs, this review aims to provide a clinically relevant overview to inform decision-making in the management of women of reproductive age receiving GLP-1RA therapy.

## 2. Materials and Methods

### 2.1. Study Design

This study was conducted as a narrative literature review to summarize current evidence on the reproductive effects and pregnancy safety of glucagon-like peptide-1 receptor agonists (GLP-1RAs), with particular emphasis on semaglutide and liraglutide. The review aimed to synthesize available pharmacological, preclinical, and clinical evidence related to fertility restoration, unintended pregnancy, and reproductive safety.

### 2.2. Search Strategy

A structured literature search was performed using the databases PubMed/MEDLINE, Embase, the Cochrane Library, and ClinicalTrials.gov. The search covered studies published between 1 January 2020 and 31 December 2025, while earlier foundational studies were retrieved when necessary to contextualize pharmacological mechanisms and regulatory recommendations. The search strategy combined controlled vocabulary (MeSH terms) and free-text keywords using Boolean operators. Core search terms included:

(GLP-1 receptor agonist OR GLP-1RA OR semaglutide OR liraglutide OR exenatide OR dulaglutide OR tirzepatide OR incretin) AND

(pregnancy OR conception OR fertility OR “polycystic ovary syndrome” OR PCOS OR ovulation OR fetal OR embryo* OR congenital OR teratogen* OR miscarriage OR “spontaneous abortion” OR preconception).

Additional keyword combinations such as “pregnancy outcomes”, “unplanned pregnancy”, and “oral contraceptive” were used to capture relevant variations in terminology. Searches were limited to English-language peer-reviewed publications.

### 2.3. Eligibility Criteria

Studies were included if they:

- Reported data on GLP-1 receptor agonists and reproductive outcomes;
- Examined fertility, ovulation, pregnancy exposure, fetal outcomes, or reproductive safety;
- Included human studies, clinical trials, observational studies, registries, pharmacovigilance reports, or relevant preclinical studies;
- Were published in peer-reviewed journals.

Studies were excluded if they:

- Did not involve GLP-1 receptor agonists;
- Did not address reproductive or pregnancy-related outcomes;
- Were non-English publications;
- Were editorials, commentaries, or studies lacking primary data relevant to reproductive outcomes.

### 2.4. Study Selection Process

The literature search identified a total of 1086 records from the selected databases (PubMed/MEDLINE, Embase, Cochrane Library, and ClinicalTrials.gov) and additional manual reference screening. After the removal of duplicate records, 102 articles remained for title and abstract screening. During this stage, 44 records were excluded because they were not directly related to GLP-1 receptor agonists and reproductive outcomes, were editorial/commentary articles, or lacked relevant clinical or experimental data. The remaining 58 articles underwent full-text assessment for eligibility. Of these, 9 articles were excluded due to insufficient reporting of reproductive outcomes, lack of direct relevance to GLP-1RA exposure in fertility or pregnancy, or publication in non-English languages. Ultimately, 49 studies met the eligibility criteria and were included in the final narrative synthesis evaluating fertility restoration, pregnancy exposure, and reproductive safety outcomes associated with GLP-1 receptor agonists.

### 2.5. Data Extraction and Evidence Synthesis

For each included study, key variables were systematically extracted into a structured evidence matrix to facilitate consistent comparison across studies. The extracted information included the study design, population characteristics, type of GLP-1 receptor agonist (GLP-1RA) used and its dosage, timing of drug exposure relative to conception or pregnancy, and the presence of comparator groups where applicable. In addition, data on reproductive outcomes, such as ovulation, menstrual regularity, and conception rates, as well as pregnancy outcomes, including miscarriage, congenital anomalies, and neonatal outcomes, were collected. The extracted evidence was then synthesized narratively, with studies categorized according to pharmacological mechanisms, preclinical evidence, clinical fertility outcomes, pregnancy exposure data, and pharmacovigilance findings. Particular attention was given to identifying consistency or divergence of findings across different study designs and to examining potential sources of heterogeneity, including underlying metabolic conditions, severity of obesity, and concomitant medication use.

Although a structured search strategy and study selection process were employed to enhance transparency, this review did not follow a formal systematic review methodology (e.g., no predefined protocol, duplicate screening, or risk-of-bias assessment), and is therefore presented as a narrative synthesis.

**Figure 1:**
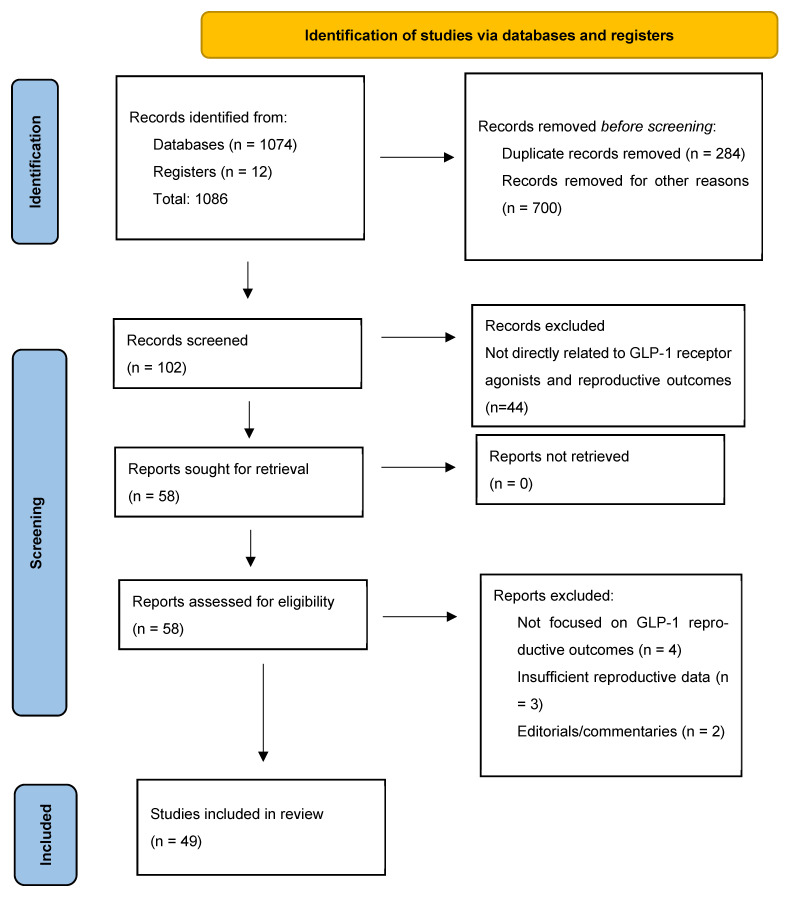
PRISMA flow diagram depicting the study selection process.

The PRISMA flow diagram of each stage of the article selection process is described in the flowchart shown in Figure 1 [19].

## 3. Results

### 3.1. Mechanistic and Pharmacokinetic Considerations

GLP-1 is an incretin hormone released by enterocytes that promotes insulin production dependent on glucose levels. GLP-1 additionally decreases glucagon release, slows gastric emptying, and enhances feelings of fullness. Pharmacologic analogues have been developed to prolong GLP-1 activity and improve its therapeutic effects on weight reduction and glycemic control. GLP-1RAs indirectly influence the hypothalamic–pituitary–gonadal axis by diminishing appetite and lowering insulin resistance. This may also contribute to the restoration of ovulatory function in women with obesity [16]. GLP-1RAs for the treatment of type 2 diabetes mellitus and chronic weight loss have been approved. In addition to reducing obesity, these agents also improve visceral and hepatic fat content, glucose metabolism, and have potential effects on reproductive function [17]. In patients with polycystic ovary syndrome (PCOS), liraglutide has been shown to reduce body weight, visceral fat, and fasting glucose, and to increase sex hormone-binding globulin (SHBG). These metabolic gains may be converted to improved fertility, but pregnancy-related safety data are also scarce [17]. GLP-1RAs are large peptide compounds, which are believed to cross the placenta only sparingly, but placental penetration may be heterogeneous, especially early in pregnancy. Long elimination half-lives, weekly dose accumulation, and delayed clearance in renal impairment raise concern about persistent fetal exposure after discontinuation [7,11]. From a pharmacokinetic standpoint, long-acting GLP-1RAs persist for weeks after the last dose (e.g., semaglutide has an elimination half-life of approximately 1 week), so clinically meaningful exposure may continue well beyond discontinuation [16]. This is the practical basis for the commonly cited preconception washout interval, discontinuing long-acting agents about 2 months before attempting conception—to reduce the likelihood of drug exposure during early organogenesis [16,18]. Accordingly, regulatory and product-label recommendations advise against GLP-1RA use during pregnancy and support discontinuation before conception when feasible.

### 3.2. Preclinical and Animal Safety

Preclinical developmental-toxicity evidence comes largely from rodent and rabbit models and regulatory product information. Across rat, mouse, and rabbit studies, maternal exposure to GLP-1RAs during gestation was associated with dose-dependent reductions in fetal weight and/or growth, delayed/irregular ossification, and skeletal variants (e.g., wavy ribs), typically occurring alongside decreased maternal food intake and reduced maternal weight gain; reduced embryonic survival was also linked to maternal weight loss [11]. In rats, semaglutide exposure produced more severe abnormalities, including visceral anomalies and major skeletal malformations [11]. In a mechanistic mouse experiment, pregnant A/J mice received a single subcutaneous dose of recombinant GLP-1 (1000 nmol/kg) either in early gestation (E1) or late gestation (E15) [12]. Both exposure windows reduced maternal weight and neonatal birthweight, and late-gestation exposure increased neonatal mortality and skin detachment [12]. Mechanistic mouse work demonstrated that maternal semaglutide exposure in late gestation reduced fetal and placental growth and downregulated placental nutrient transport systems, reinforcing fetal growth restriction as a plausible class-related risk at pharmacologic exposure in pregnancy [13]. Separately, reproductive-axis effects have been reported outside pregnancy: in adult female rats treated with subcutaneous liraglutide for 4 weeks using a weekly dose-escalation regimen (0.6, 1.2, 1.8, then 2.4 mg/kg/day), investigators observed reduced LH/FSH with decreased ovarian estrogen/progesterone receptor expression, increased granulosa-cell apoptosis, and uterine inflammatory infiltration; partial recovery was described after a 2-week drug-free discontinuation period [15] Placental-transfer studies using ex vivo perfusion and trophoblast models found very low maternal-to-fetal transfer of dulaglutide (≈0.2–0.7% at term) and negligible transfer early in gestation, suggesting limited fetal exposure for large peptide GLP-1RAs, although caution remains warranted [20]. Interpretation should also consider semaglutide dose and indication. Semaglutide is used at different maintenance doses according to indication, including 1.0 mg/week in clinical type 2 diabetes populations and 2.4 mg once weekly as an approved chronic weight-management regimen. Given that the preclinical GLP-1RA developmental-toxicity findings summarized in this review are dose-dependent (e.g., reduced fetal growth, ossification changes, and skeletal variants, commonly alongside maternal weight loss) [11], and that mechanistic work has demonstrated semaglutide-associated reductions in fetal and placental growth with altered placental nutrient transport in late gestation models [13], interpretation of inadvertent exposure should explicitly consider dose, duration, and timing relative to organogenesis. This dose–exposure framework supports indication-specific preconception counseling and discontinuation planning for individuals attempting conception [16,18].

### 3.3. Fertility Effects and Reproductive Implications

The interplay between obesity, insulin resistance, and female infertility is well-established. In obesity and type 2 diabetes, women frequently develop chronic anovulation, hyperandrogenism, and subfertility, partly through disruption of the hypothalamic–pituitary–ovarian axis. In this context, GLP-1 receptor activity has been identified in the hypothalamus, pituitary, and ovarian granulosa cells, supporting possible effects of GLP-1 signaling on gonadotropin secretion and follicular growth. Within the ovary, GLP-1RAs may reduce granulosa-cell apoptosis and promote follicular development, suggesting a plausible direct reproductive effect [17].

Clinical studies in women with overweight/obesity and PCOS also suggest reproductive benefit alongside metabolic improvement. Exenatide once weekly combined with metformin produced greater reductions in weight, waist circumference, insulin resistance, and triglycerides while improving androgen-related parameters compared with metformin alone [21]. Metformin plus liraglutide improved hyperandrogenemia more effectively than metformin alone and was associated with stronger improvements in LH and progesterone profiles [22]. A pilot randomized trial found that beinaglutide plus metformin produced greater reductions in BMI and improved insulin resistance compared with metformin alone in obese women with PCOS, supporting class-wide reproductive-metabolic benefit signals beyond semaglutide and liraglutide [23]. Abdalla et al. reported in a systematic review of incretin-based therapies for PCOS that GLP-1RAs and DPP-4 inhibitors were associated with reductions in body weight, fasting insulin, and androgen levels, together with increased SHBG [24]. One randomized trial within that review reported a spontaneous pregnancy rate of 43.6% with exenatide versus 18.7% with metformin after 12 weeks [24]. Similar improvements in weight loss, insulin sensitivity, menstrual regularity, and spontaneous conception have also been reported in PCOS cohorts [25]. Overall, GLP-1RAs may improve the metabolic milieu associated with PCOS by reducing body weight, visceral adiposity, hyperinsulinemia, and androgen excess, changes that may improve ovulatory function and endometrial receptivity, although the extent to which these benefits reflect direct reproductive effects versus secondary metabolic improvement remains uncertain [17,21,22,23,24,25].

In women, particularly those with obesity and PCOS, pooled clinical evidence suggests that GLP-1RAs improve metabolic dysfunction together with reproductive endpoints, including menstrual regularity and ovulation, and in some studies are associated with higher rates of spontaneous conception than metformin-based approaches [14,26,27]. In men with obesity-related reproductive dysfunction, emerging clinical evidence suggests that GLP-1RA-supported metabolic improvement and weight-loss maintenance may be accompanied by improvements in testosterone and semen parameters, although human data remain heterogeneous and occasional reversible adverse case reports exist [28,29,30].

**Figure 2:**
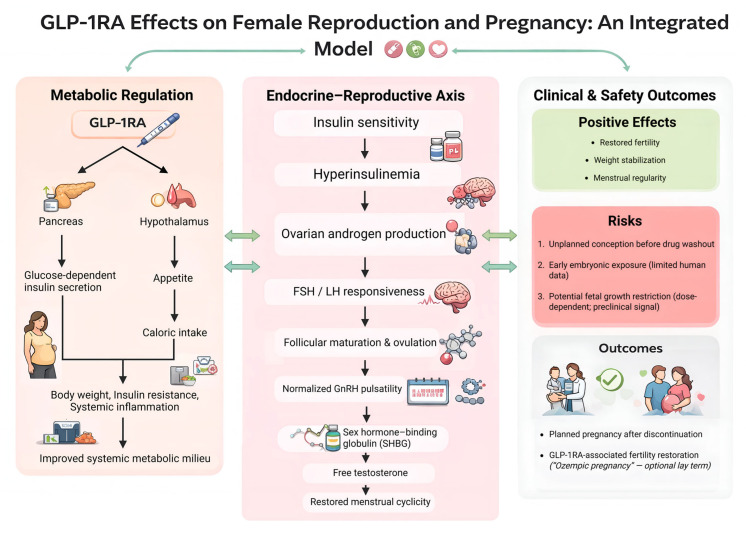
Integrated model of GLP-1RA effects on female reproduction and pregnancy risk. Conceptual framework linking GLP-1RA-mediated metabolic regulation (appetite, caloric intake, insulin secretion) to downstream endocrine-reproductive effects (insulin sensitivity, ovarian androgen production, gonadotropin responsiveness, follicular maturation/ovulation, SHBG, and free testosterone) and clinical outcomes. The model also depicts key risk mechanisms for unintended pregnancy, including rapid fertility restoration, incomplete washout prior to conception, limited human pregnancy safety data, and a preclinical signal for fetal growth effects. Abbreviations: FSH, follicle-stimulating hormone; LH, luteinizing hormone; GnRH, gonadotropin-releasing hormone; SHBG, sex hormone-binding globulin; GLP-1RA, glucagon-like peptide-1 receptor agonist. This figure is an original conceptual framework developed by the authors to synthesize mechanistic and reproductive themes from the reviewed literature.

As shown in Figure 2, GLP-1RAs interact with metabolic and neuroendocrine pathways that may support reproductive function while also intersecting with pregnancy-related safety considerations. Histological studies demonstrate GLP-1 receptor expression in reproductive tissues, including the pituitary, ovaries, and endometrium, supporting a physiologic role in fertility [31]. Preclinical models suggest that GLP-1RAs may reduce ovarian inflammation, lower androgen excess, normalize cytokine profiles, and increase SHBG, changes that may contribute to improved menstrual cyclicity in women with PCOS [31,32]. However, despite these favorable metabolic and hormonal effects, evidence remains insufficient to determine whether GLP-1RAs exert direct effects on gonadotropin signaling, follicular development, or hormone secretion independent of weight loss [17].

### 3.4. Human Pregnancy Exposure, Unintended Conception, and Clinical Implications

Human evidence regarding GLP-1RA exposure in pregnancy remains limited and is derived largely from regulatory datasets, observational cohorts, registries, and case reports. Based on FDA/EMA data, Parker et al. reported 164 unplanned pregnancies among approximately 32,000 GLP-1RA-treated women, with outcomes including 43% live births, 22% spontaneous abortions, and 2.7% congenital anomalies, comparable to the placebo in that dataset [6]. Kolding et al. examined 104,422 Danish pregnancies, including 32 with first-trimester semaglutide exposure, and found no increase in major malformations [8]. In a nationwide Taiwanese cohort of women with pregestational type 2 diabetes, periconceptional GLP-1RA exposure was not associated with increased risk of major congenital malformations or other adverse outcomes compared with insulin exposure, although residual confounding by indication remained an important limitation [9]. Collectively, these data do not identify a clear teratogenic signal, but sample sizes remain small and interpretation is constrained by confounding from diabetes, obesity, and associated comorbidities [6,8,9,15]. Table 1 summarizes the principal human studies included in this review.

Case reports and small series provide limited but clinically relevant additional information. Early semaglutide exposure was described with normal outcome in one report, while high-dose liraglutide exposure was associated with low birth weight but no structural malformations in another [33,34]. Varughese et al. described inadvertent exposures with generally reassuring neonatal outcomes, although such reports remain anecdotal [7]. A case report of continued dulaglutide exposure until 34 weeks of pregnancy described delivery of a neonate without congenital anomalies, though transient neonatal hypoglycemia requiring brief intravenous glucose support was reported [35]. Two sibling pregnancies conceived during exenatide therapy were also reported; treatment was discontinued during the first trimester, one infant had an atrial septal defect that closed spontaneously, and the second was healthy at term [36].

**Figure 3:**
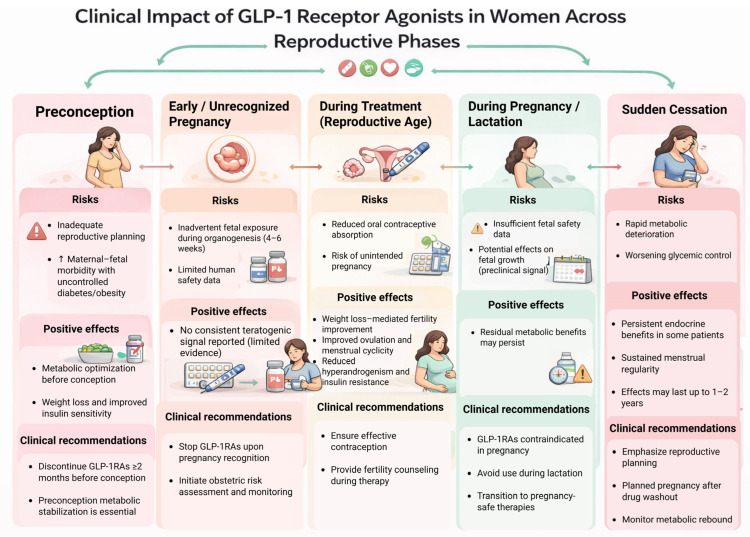
Clinical impact of GLP-1RAs across reproductive phases. Schematic summary of key risks, potential benefits, and clinical recommendations for GLP-1RA use across (i) preconception, (ii) early/unrecognized pregnancy, (iii) treatment during reproductive age, (iv) pregnancy/lactation, and (v) sudden cessation. The figure highlights risk points for inadvertent fetal exposure during early organogenesis, potential reductions in oral contraceptive absorption, and the importance of reproductive planning, contraception, metabolic stabilization, and discontinuation prior to conception. Abbreviations: GLP-1RA, glucagon-like peptide-1 receptor agonist. This figure is an original conceptual clinical framework developed by the authors to summarize stage-specific management considerations and is intended as a visual aid rather than a quantitative evidence model.

Real-world use of GLP-1RAs in women of reproductive age is increasing. General practice data from Australia demonstrated a rapid rise in prescribing in reproductive-aged women, with increasing overlap between GLP-1RA initiation and contraceptive use [37]. Ahrens et al. also reported increasing prescription rates before and after pregnancy [2]. Conference data from Hviid et al. described similar distributions of obstetric outcomes while emphasizing the need for centralized pregnancy registries [38]. In the InPreSS consortium, Cesta et al. evaluated more than 50,000 pregnancies in women with pregestational type 2 diabetes and found no increased risk of major congenital malformations after periconceptional exposure to GLP-1RAs or other second-line noninsulin agents compared with insulin, although prescribing around conception was increasing over time [1]. Together, these findings indicate that GLP-1RA exposure around conception is becoming more common in routine practice, while the corresponding human safety evidence remains limited. Figure 3 presents a phase-based conceptual framework for GLP-1RA use across the reproductive timeline.

An important clinical consequence of GLP-1RA therapy is that restoration of ovulation may increase the likelihood of unintended conception, especially in women with obesity and PCOS. This possibility is amplified by the high baseline rate of unplanned pregnancy in women with obesity and diabetes [39,40,41]. In this context, inadvertent exposure may occur during early organogenesis, when pregnancy is still unrecognized [40]. Thus, GLP-1RA-associated fertility restoration creates a clinical paradox in which reproductive benefit is accompanied by uncertainty regarding early pregnancy exposure [10,24]. In lay and media discourse, this phenomenon has sometimes been described as “Ozempic pregnancy”; however, the available evidence supports fertility restoration and an increased opportunity for conception rather than a proven drug-specific increase in pregnancy rates [31,37,42,43].

**Figure 4:**
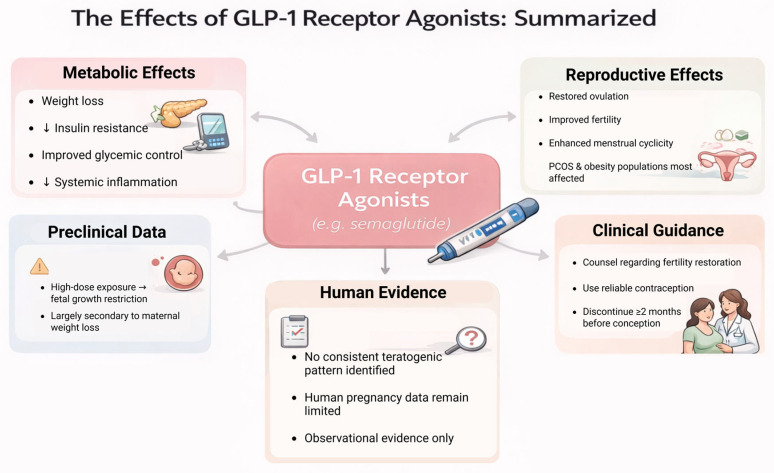
Summary of GLP-1RA effects relevant to reproductive safety. Overview of metabolic effects (weight loss, reduced insulin resistance, improved glycemic control, reduced systemic inflammation), reproductive effects (restored ovulation, improved fertility, enhanced menstrual cyclicity—particularly in PCOS/obesity), and the evidence base informing reproductive safety (preclinical findings at high doses, limited human pregnancy data). The figure highlights clinical counseling priorities including reliable contraception, counseling regarding fertility restoration, and discontinuation before planned conception. Abbreviations: GLP-1RA, glucagon-like peptide-1 receptor agonist; PCOS, polycystic ovary syndrome. This figure is an original conceptual synthesis developed by the authors to summarize clinical themes emerging from the reviewed evidence and does not represent a formal quantitative evidence model.

An additional under-discussed clinical issue is maternal volume status and renal perfusion during inadvertent semaglutide exposure. Semaglutide prescribing information warns that gastrointestinal adverse reactions may lead to dehydration and acute kidney injury, and that renal monitoring is advised when clinically significant nausea, vomiting, or diarrhea occur [44]. In pregnancy, this issue may be especially relevant because early gestation is often accompanied by nausea, reduced oral intake, and fluid shifts. Indirect contextual evidence also suggests that women with baseline dysglycemia may be more vulnerable to renal stress; for example, female kidney donors with prediabetes have shown higher post-donation proteinuria and higher systolic and diastolic blood pressure than donors without diabetes [45]. These concerns do not establish a specific signal of fetal renal toxicity, but they reinforce the need for careful interpretation of inadvertent exposure in the setting of limited human data [44,45]. As summarized in Figure 4, GLP-1RA therapy in women of reproductive age is characterized by the coexistence of metabolic benefit, fertility restoration, and uncertainty regarding unintended early pregnancy exposure.

## 4. Discussion

### 4.1. Fertility Effects and the Question of Direct Versus Indirect Benefit

This review highlights a clinically important dual effect of GLP-1RAs in reproductive-aged patients: metabolic improvement with potential fertility restoration, especially in obesity/PCOS, together with greater opportunity for unintended periconception exposure. Interventional studies and pooled analyses in PCOS consistently show improvements in weight and insulin resistance alongside reproductive endpoints (menstrual regularity, ovulation), with some trials reporting higher spontaneous conception rates compared with metformin-based approaches [14,21,22,23,24,25,26,27]. For example, one randomized trial reported spontaneous pregnancy rates of 43.6% with exenatide versus 18.7% with metformin after 12 weeks [21,24].

An important interpretive distinction is whether reproductive changes observed with GLP-1RAs reflect direct receptor-mediated effects or secondary benefits from weight loss and improved insulin resistance. Preclinical and mechanistic data support plausible direct actions on the hypothalamus, pituitary, ovarian granulosa cells, gonadotropin signaling, and follicular dynamics [17,46]. However, most human improvements in menstrual regularity, ovulation, and spontaneous conception have been demonstrated in women with obesity or PCOS in parallel with reductions in body weight, visceral adiposity, fasting insulin, and hyperandrogenism [14,21,22,23,24]. Thus, the clinical fertility signal in humans is best interpreted as mixed rather than purely direct, with weight loss and metabolic normalization likely accounting for a substantial proportion of the observed benefit. This distinction is equally important when considering pregnancy safety: adverse developmental signals in animal studies often occurred together with maternal weight loss and reduced food intake, complicating attribution to direct embryotoxicity alone [11,12,13].

### 4.2. Human Pregnancy Exposure Data and Interpretation of Safety Signals

Population prescribing studies indicate that GLP-1RA exposure before and around pregnancy is increasing, including in general practice cohorts of reproductive-aged women and in statewide pregnancy datasets [2,37]. In women with obesity and diabetes, who already have an elevated baseline risk of unplanned pregnancy and adverse obstetric outcomes, this trend increases the likelihood of exposure during early organogenesis, particularly when pregnancy is unrecognized or treatment discontinuation is delayed [37,38].

Preclinical evidence across rodent and rabbit models, as well as mechanistic mouse studies, suggests dose-dependent reductions in fetal growth, delayed or irregular ossification, and skeletal variants, often alongside decreased maternal intake and maternal weight loss [11,12,13]. Mechanistic late-gestation semaglutide exposure has also been shown to reduce fetal and placental growth and downregulate placental nutrient transport systems, reinforcing fetal growth restriction as a biologically plausible class-related concern at pharmacologic exposure [13]. At the same time, placental-transfer studies of large peptide GLP-1RAs such as dulaglutide suggest very low maternal-to-fetal transfer at term, indicating that fetal exposure may be limited for some agents, although this does not eliminate concern given inter-agent differences and uncertainty in early gestation [20].

The human evidence summarized in this review is cautiously reassuring but remains underpowered for rare outcomes. Regulatory pharmacovigilance summaries, observational cohorts, and registry-based analyses have not identified a consistent pattern of congenital anomalies after early GLP-1RA exposure, but these datasets remain limited by small exposed sample sizes, confounding by indication, heterogeneous exposure definitions, and incomplete capture of early pregnancy loss [6,7,8,9,10,15]. Accordingly, the absence of a clear teratogenic signal should not be interpreted as evidence of safety.

At the molecule-specific level, the reproductive evidence is uneven across the GLP-1RA class. Semaglutide accounts for much of the current concern regarding inadvertent early-pregnancy exposure because of its widespread use and prolonged elimination, which makes preconception washout especially relevant [8,16,33,47]. Liraglutide and exenatide have comparatively more established data in women with obesity and PCOS for metabolic and reproductive improvement, but pregnancy-exposure data for these agents remain limited and are derived mainly from observational evidence and case reports [21,22,34,36]. Dulaglutide has even less pregnancy-related evidence, relying largely on placental-transfer studies and isolated case experience [20,35]. Tirzepatide is clinically notable for a different reason: its reproductive relevance currently lies more in its effect on oral contraceptive exposure during treatment initiation and dose escalation than in pregnancy-outcome data [48,49]. GLP-1RAs should therefore not be interpreted as a fully uniform class across all reproductive questions.

### 4.3. Clinical Counseling Implications: Contraception, Washout, and Multidisciplinary Care

In women of reproductive age receiving GLP-1RA therapy, pregnancy intention and contraception planning should be addressed at treatment initiation and revisited during dose escalation [14,41]. Current evidence also suggests that counseling remains inconsistently delivered in routine care, with pregnancy-risk discussions and contraceptive guidance still under-documented in women receiving metabolic therapy [42,43,50].

Practical counseling should also account for treatment-related factors that may affect contraceptive reliability or preconception planning. Professional guidance indicates that persistent vomiting or diarrhea may compromise oral contraceptive effectiveness, and that tirzepatide may specifically reduce oral contraceptive exposure during treatment initiation and dose escalation, supporting temporary backup or non-oral contraception in that setting [48,49]. In addition, early semaglutide exposure in pregnancy has often occurred without adequate preconception counseling and has been associated with poorer glycemic control [33]. Lin et al. also reported mild gastrointestinal and neurologic adverse effects, including nausea, vomiting, and dizziness, which may further complicate treatment during the periconception period [51]. Varughese et al. emphasized that counseling should begin at GLP-1RA initiation and continue throughout the preconception period, while also addressing the risks of premature discontinuation and loss of metabolic benefit [7].

For patients actively planning conception, current product-label and clinical-guidance-based recommendations generally support the discontinuation of long-acting GLP-1RAs such as semaglutide in advance of conception in order to reduce the likelihood of exposure during early organogenesis [4,15,16,41,52]. Because this can be complicated by weight regain or metabolic deterioration after treatment cessation, preconception planning should include an explicit transition strategy addressing nutrition, glycemic management, and weight maintenance [4,16,41,53,54]. For selected nonpregnant individuals discontinuing GLP-1RA therapy, structured meal-timing approaches such as time-restricted eating may offer a supervised interim metabolic strategy, although these approaches should not be extrapolated to pregnancy itself [55,56,57,58].

If inadvertent pregnancy exposure occurs, management should prioritize prompt medication review, assessment of hydration status and concomitant nephrotoxic or teratogenic risks, and individualized follow-up rather than reflexive reassurance or alarm [6,7,41,44]. Because the evidence base remains limited and pregnancy exposures are often identified late, multidisciplinary care involving endocrinology, obstetrics, maternal–fetal medicine, and, when appropriate, reproductive medicine is likely to provide the safest framework for medication transition and surveillance [6,11,41].

### 4.4. Strengths and Limitations of the Available Evidence

The current evidence base remains limited and should be interpreted cautiously. Fertility-related benefits are supported mainly by small interventional studies in women with obesity or PCOS, often with short follow-up and concurrent metabolic improvement, making it difficult to separate direct pharmacologic reproductive effects from indirect benefits mediated by weight loss, improved insulin sensitivity, and reduced hyperandrogenism [14,17,21,22,23,24,32]. Pregnancy-safety data are even more limited, relying largely on inadvertent exposure captured through registries, pharmacovigilance reports, observational cohorts, and case reports rather than on prospectively designed pregnancy studies [2,6,8,10,38]. These studies are vulnerable to confounding by indication, small exposed sample sizes, heterogeneous exposure definitions, incomplete capture of early pregnancy loss, and variable adjustment for glycemic control and concomitant medications [6,7,9]. In addition, the strength of evidence differs across individual GLP-1RAs, so class-wide conclusions may obscure important molecule-specific differences in exposure duration, washout considerations, and contraceptive counseling implications [4,16,35,48,49]. Preclinical studies provide important biologic safety signals, but translation to human pregnancy remains limited by species differences, dose comparability, and the frequent coexistence of maternal weight loss and reduced food intake [11,12,13]. Because randomized pregnancy-exposure trials are unlikely to be feasible, future progress will depend on large prospective multicenter registries with standardized definitions of exposure timing and maternal, fetal, and neonatal outcomes [10,18,58]. Importantly, the absence of a consistent teratogenic signal in the currently available human data should not be interpreted as evidence of safety, but rather as evidence that remains underpowered and vulnerable to confounding.

### 4.5. Future Directions

Given ethical constraints on randomized pregnancy-exposure studies, improved inference will depend on harmonized pregnancy registries, standardized endpoints including miscarriage, fetal growth, congenital anomalies, and neonatal outcomes, and careful stratification by drug, dose, and timing of exposure [10,18]. Prescribing-trend data support the urgency of implementing these systems as real-world exposure becomes more common [2,37]. In addition, future work should clarify molecule-specific differences in reproductive safety, contraceptive interactions, and pharmacokinetic washout intervals, particularly for newer agents such as tirzepatide [48,49,59,60]. At a systems level, more consistent integration of preconception counseling and pharmacovigilance pathways into routine metabolic care may help reduce unintended exposure while improving the quality of future real-world evidence [61,62,63].

## 5. Conclusions

The increasing use of GLP-1 receptor agonists among women of reproductive age has created an important clinical and public health issue at the intersection of metabolic therapy and reproductive care. These agents may improve weight, insulin resistance, and fertility, particularly in women with obesity and PCOS, but such benefits may also increase the likelihood of unintended conception if contraception and preconception planning are not adequately addressed. Current human pregnancy safety data remain limited to small observational cohorts, pharmacovigilance reports, and case studies, all of which are subject to confounding and incomplete outcome ascertainment. Therefore, the absence of a consistent teratogenic signal should not be interpreted as confirmation of safety. Clinicians must balance therapeutic benefit with reproductive caution by ensuring that women of reproductive age receiving GLP-1 receptor agonists are provided with individualized counseling, effective contraception, and a clear plan for discontinuation before planned conception. Strengthened pharmacovigilance systems and large, well-designed pregnancy registries will be essential to improve the evidence base and guide future recommendations. Overall, the growing possibility of unintended conception during GLP-1 receptor agonist therapy highlights the need to integrate reproductive planning into long-term metabolic management through a proactive multidisciplinary approach.

## Figures and Tables

**Table 1:** Selected human studies on GLP-1RA therapy relevant to fertility restoration, pregnancy exposure, and reproductive counseling, including study design, population size, and key limitations.

Study (Ref)	Design/n	Context	Key Finding	Limitation/Evidence Note
Ma et al. [21]	Randomized trial; n = 50	Exenatide + metformin vs. metformin in overweight/obese PCOS	Greater improvement in weight, insulin resistance, and androgen-related parameters	Small, short-term fertility study
Xing et al. [22]	Randomized trial; n = 60	Liraglutide + metformin vs. metformin in overweight PCOS	Better improvement in hyperandrogenemia and reproductive hormone profile	Moderate sample, short follow-up
Wen et al. [23]	Pilot randomized trial; n = 64	Beinaglutide + metformin vs. metformin in obese PCOS	Greater reduction in BMI and insulin resistance	Pilot study, limited duration
Abdalla et al. [24]	Evidence synthesis; 14 interventional + 2 case-control studies	Incretin-based therapies in PCOS	Reported metabolic and reproductive improvement; one trial showed higher spontaneous pregnancy with exenatide	Secondary evidence; heterogeneous studies
Voros et al. [14]	Systematic review	GLP-1RAs in reproductive health	Signal for improved menstrual regularity, ovulation, and spontaneous conception in selected populations	Secondary evidence; mixed study quality
Parker et al. [6]	Regulatory safety dataset; 164 pregnancies among ~32,000 treated women	Inadvertent pregnancy during GLP-1RA trials	No clear teratogenic signal in available trial safety data	Not a designed pregnancy study
Kolding et al. [8]	Nationwide cohort; 104,422 pregnancies, 32 semaglutide-exposed	First-trimester semaglutide exposure	No increase in major malformations	Very small exposed subgroup
Chou et al. [9]	Nationwide cohort	Periconceptional GLP-1RA exposure in pregestational T2D	No increased risk of major congenital malformations vs. insulin	Residual confounding by indication
Dao et al. [10]	Prospective multicenter cohort; n = 168 exposed	Early pregnancy GLP-1RA exposure	No strong teratogenic signal observed	Underpowered for rare outcomes
Morton et al. [33]	Case report; n = 1	First-trimester semaglutide	Normal outcome reported	Anecdotal evidence only
Abushanab et al. [34]	Case report; n = 1	High-dose liraglutide exposure	Low birth weight, no structural malformation	Anecdotal evidence only
Molteni et al. [35]	Case report; n = 1	Dulaglutide until 34 weeks	No congenital anomaly; transient neonatal hypoglycemia	Anecdotal evidence only
Doğan et al. [36]	Case series; n = 2	First-trimester exenatide exposure	One ASD resolved spontaneously; one healthy term infant	Very small case series
Thapaliya et al. [37]	Retrospective cohort	Prescribing in reproductive-aged women	Rising GLP-1RA use and overlap with contraceptive use	Exposure trend only, not outcome study
Ahrens et al. [2]	Statewide observational study	GLP-1RA exposure before/during/after pregnancy	Rising exposure prevalence around pregnancy windows	Exposure trend only
Hviid et al. [38]	Observational abstract	Early pregnancy liraglutide exposure	No major increase in complications reported	Abstract-level evidence

## Data Availability

No new data were created or analyzed in this study. Data sharing is not applicable to this article.

## Abbreviations and Glossary

ADMs	Antidiabetic Medications
A/J	A/J Mouse Strain
ART	Assisted Reproductive Technology
BMI	Body Mass Index
CEU	Clinical Effectiveness Unit
DPP-4	Dipeptidyl Peptidase-4
E1	Embryonic Day 1 (Early Gestation)
E15	Embryonic Day 15 (Late Gestation)
EMA	European Medicines Agency
FDA	Food and Drug Administration
FSH	Follicle-Stimulating Hormone
FSRH	Faculty of Sexual and Reproductive Healthcare
GI	Gastrointestinal
GIP	Glucose-Dependent Insulinotropic Polypeptide
GLP-1	Glucagon-Like Peptide-1
GLP-1RA/GLP-1RAs	Glucagon-Like Peptide-1 Receptor Agonist(s)
GnRH	Gonadotropin-Releasing Hormone
HPO Axis	Hypothalamic–Pituitary–Ovarian Axis
IVF	In Vitro Fertilization
LH	Luteinizing Hormone
MeSH	Medical Subject Headings
MFM	Maternal–Fetal Medicine
PCOS	Polycystic Ovary Syndrome
SHBG	Sex Hormone-Binding Globulin
SG	Semaglutide
T2D	Type 2 Diabetes
Hypothalamic–Pituitary–Ovarian (HPO) Axis	The hormonal system linking the hypothalamus, pituitary, and ovaries that regulates ovulation, menstruation, and fertility.
Polycystic Ovary Syndrome (PCOS)	A hormonal disorder causing chronic anovulation, high androgen levels, and ovarian cysts, leading to infertility and metabolic imbalance.
Ovulatory Dysfunction	Irregular or absent ovulation caused by hormonal disturbances, often seen in PCOS and obesity.
Endometrial Receptivity	The phase when the uterine lining becomes optimal for embryo implantation.
Oocyte Quality	The developmental potential of the egg, influenced by chromosomal and mitochondrial integrity.
Gonadotropins (FSH & LH)	Pituitary hormones that stimulate follicle growth (FSH) and trigger ovulation (LH).
Follicular Growth	The maturation of ovarian follicles preparing for ovulation under hormonal influence.
Granulosa Cells	Cells surrounding the egg that produce estrogen and support follicle development.
Sex Hormone-Binding Globulin (SHBG)	A protein that binds sex hormones, reducing free testosterone and regulating hormonal balance.
Hyperandrogenism	Elevated androgen levels in females causing hirsutism, acne, and menstrual irregularities.
Insulin Resistance	A condition where cells respond poorly to insulin, linked to PCOS, obesity, and diabetes.
Luteinizing Hormone (LH) Pulsatility	The rhythmic pattern of LH release that regulates ovulation and menstrual cycles.
Gonadotropin-Releasing Hormone (GnRH)	A hormone from the hypothalamus that stimulates FSH and LH release from the pituitary.
Endocrine Crosstalk	The interaction between different hormonal systems, such as insulin and reproductive hormones.
Teratogenicity	The potential of a substance to cause fetal malformations or developmental defects.
Periconceptional Period	The critical window around conception (about 3 months before and after fertilization).
Organogenesis	The stage of early fetal development (weeks 3–8) when organs form and teratogenic risk is highest.
Pregnancy Registry	A database tracking pregnancies exposed to specific drugs to assess safety outcomes.
Preconception Counseling	Clinical advice before conception to improve maternal health and minimize pregnancy risks.
Macrosomia	Excessive fetal growth (birth weight >4000 g), often linked to maternal diabetes.
Preterm Birth	Delivery before 37 weeks of gestation, often associated with metabolic or medication-related risks.
Spontaneous Abortion	Unintended pregnancy loss before 20 weeks of gestation.
Embryotoxicity	Toxic effects on the developing embryo that may halt development or cause defects.
Placental Transfer	Movement of drugs or substances from the maternal bloodstream to the fetus via the placenta.
Washout Period	Time needed for a medication to clear from the body before conception or another treatment.
Endocrine Disruption	Interference with normal hormone signaling affecting reproduction and development.
Maternal–Fetal Medicine (MFM)	A subspecialty of obstetrics focused on managing high-risk pregnancies.
Multidisciplinary Care	Coordinated management involving obstetricians, endocrinologists, and other specialists.
Incretin Effect	Enhanced insulin secretion after oral glucose intake, mediated by hormones like GLP-1 and GIP.
Metabolic Rebound	The worsening of metabolic parameters (like weight or glucose levels) after stopping therapy.
